# A bacosides containing *Bacopa monnieri* extract alleviates allodynia and hyperalgesia in the chronic constriction injury model of neuropathic pain in rats

**DOI:** 10.1186/s12906-017-1807-z

**Published:** 2017-06-05

**Authors:** Muhammad Shahid, Fazal Subhan, Nisar Ahmad, Ihsan Ullah

**Affiliations:** 1 Department of Pharmacy, University of Peshawar, Peshawar, Khyber Pakhtunkhwa 25120 Pakistan; 2 Department of Pharmacy, Sarhad University of Science and Information Technology, Peshawar, Pakistan; 3 Department of Pharmacy, University of Swabi, Swabi, Pakistan

**Keywords:** *Bacopa monnieri*, Bacoside-A, Rat model of neuropathy, Nociception, Pain paradigms

## Abstract

**Background:**

The current therapy of neuropathic pain is inadequate and is limited by the extent of pain relief and the occurrence of dose dependant side effects. Insufficient control of pain with conventional medications prompts the use of complementary and alternative medicine therapies by patients with neuropathic pain. This study therefore investigated a standardized methanolic extract of *Bacopa monnieri*, a widely reputed nootropic plant, for prospective antinociceptive effect in the chronic constriction injury (CCI) model of neuropathic pain.

**Methods:**

Placement of four loose ligatures around the sciatic nerve produced partial denervation of the hindpaw in rats. *Bacopa monnieri* (40 and 80 mg/kg, p.o) and the positive control, gabapentin (75 mg/kg, i.p), were administered daily after CCI or sham surgery and the behavioral paradigms of static- and dynamic-allodynia (paw withdrawal threshold to von Frey filament stimulation [PWT] and paw withdrawal latency to light-brushing [PWL]), cold-allodynia (paw withdrawal duration [PWD] to acetone), heat- (PWL to heat-stimulus) and punctate-hyperalgesia (PWD to pin-prick) were assessed on days 3, 7, 14 and 21.

**Results:**

CCI consistently generated static- (days 3–21), dynamic- (days 14–21) and cold-allodynia (days 3–21) plus heat- and mechano-hyperalgesia (days 3–21). The tested doses of *Bacopa monnieri* significantly attenuated the CCI-induced allodynia and hyperalgesia, exemplified by increased PWT (days 7–21), PWL to light brushing (days 14–21) and heat (days 7–21) as well as decreased PWD to pin prick and cold stimuli (days 3–21). The extract also counterbalanced the CCI-induced aberrations in the nociceptive behaviors by increasing the pain threshold to that of pre-surgery baseline. Gabapentin also afforded analogous beneficial behavioral profile but of higher magnitude.

**Conclusions:**

Our findings suggest that *Bacopa monnieri* can be used as adjuvant therapy for neuropathic pain conditions afflicted with allodynia and hyperalgesia.

**Electronic supplementary material:**

The online version of this article (doi:10.1186/s12906-017-1807-z) contains supplementary material, which is available to authorized users.

## Background

Neuropathic pain refers to pain that originates from pathology of the nervous system. Its symptoms include spontaneous and stimulus evoked painful sensations manifested as paraesthesia, paroxysmal pain, hyperalgesia (pain sensation is significantly enhanced) or allodynia (non-noxious stimuli cause pain) [1]. Neuropathic pain is a disease of global burden. Despite rapid development of neuroscience and modern techniques related to drug discovery, effective drugs to mitigate the symptoms of neuropathic pain are still lacking. A plethora of randomized controlled trials have demonstrated potential effectiveness of gabapentinoids, tricyclic antidepressants and opioids; however, these pain ameliorating medications are inadequate and their effectiveness is limited by the extent of pain relief provided and the occurrence of significant side effects [2].

Complementary and alternative medicine therapies are regularly used by patients with chronic neurological disorders including neuropathic pain. The most common reason for their usage is insufficient control of pain with conventional medications [3]. Among a large number of plant derived remedies, *Bacopa monnieri* (Linn.) Pennell [family: *Scrophulariaceae*] has been considered as a reputable nootropic medicine and is traditionally used for memory enhancing, rejuvenating, increasing longevity and promoting progeny [4]. Experimental studies have shown that *Bacopa monnieri* possesses anti-dementia [5], cognitive enhancing [6, 7], antidepressant [8], anti-anxiety [9], antiepileptic [10–12], antiparkinson [13, 14], anti-amnesic [15–17], antibacterial [18], antidiabetic [19], anti-inflammatory [20–22], anti-arthritis [23], antihypertensive [24], anticancer [25], anti-asthmatic [26], spasmolytic [27], antiulcer [28, 29], analgesic [30–32], anti-aging [33], antifungal [34], antioxidant [35–37], adaptogenic [38, 39], anti-addictive [40, 41], lessened narcotics-induced toxicity [42], neuroprotective [35, 43–45], antiemetic [46, 47], cardioprotective [48, 49], hepatoprotective [50–53] and nephroprotective [53, 54] properties. Clinical trials have shown that *Bacopa monnieri* has both acute [55, 56] and chronic [55–59] cognitive enhancing effects and improves memory performance in older persons [60, 61] and patients with Alzheimer’s disease [62]. The major chemical constituents isolated from *Bacopa monnieri* are dammarane type triterpenoid saponins with jujubogenin and pseudojujubogenin as the aglycones including bacosides A_1_-A_3_, bacopasaponins A-G and bacopasides I-V [63, 64]. Bacoside-A along with bacopaside-I constituted more than 96% *w*/w of the total saponins of *Bacopa monnieri* [65].

*Bacopa monnieri* has shown its utility against tonic visceral chemically-induced nociceptive pain and acute phasic thermal nociception in animal studies [66]. Additionally, *Bacopa monnieri* attenuates opioid tolerance, enhances opiates-induced analgesia and has morphine like analgesic effect without producing any tolerance to its own analgesic effect [66, 67]. However, there is little evidence of its efficacy in attenuating neuropathic pain conditions. A previous study in rats shows that *Bacopa monnieri* is an effective analgesic in a model of diabetic neuropathy as it allays streptozotocin-induced hyperalgesia and the protection has been afforded via stimulation of adenosine A_1_-receptors [68]. In this study we further corroborated the effectiveness of *Bacopa monnieri* against neuropathic pain in a well characterized rat model of chronic sciatic nerve constriction injury using the well known behavioral testing paradigms of allodynia and hyperalgesia.

## Methods

### Drugs and chemicals

Gabapentin (supplied by Lowitt Pharmaceuticals, Peshawar, Pakistan), HPLC grade acetonitrile and methanol (Sigma-Aldrich, St. Louis, USA), HPLC standards of bacoside-A_3_, bacopaside-II and bacopasaponin-C (gifted by Prof. Dr. Ikhlas Khan, National Center for Natural Products Research, School of Pharmacy, The University of Mississippi, Mississippi, USA), xylazine (Xylaz®, 20 mg/mL, Bladel, Netherlands) and ketamine (Ketarol®, 50 mg/mL, Global Pharmaceuticals, Islamabad, Pakistan).

### Preparation of *Bacopa monnieri* extract

Naturally grown *Bacopa monnieri* was collected in April from a stream near Margalla hills, Islamabad, Pakistan. After authentication by Prof. Dr. Mohammad Ibrar (Pharmacognosist; Department of Botany, University of Peshawar), a specimen was deposited in the herbarium with a voucher number 20016 (PUP). The aerial parts were separated, shade dried and coarsely grinded. The powdered material was treated with *n*-hexane and further treated with acetone to remove chlorophyll type pigments. It was subjected to extraction with methanol in a Soxhlet apparatus. The obtained extract was then filtered and concentrated in a rotary evaporator under reduced pressure at 50 °C. It was finally dried on a water bath to obtain a semisolid mass (yield, 6.5%).

### HPLC quantification of bacosides in *Bacopa monnieri*

*Bacopa monnieri* was quantified for bacoside “A” major components including bacoside-A_3_, bacopaside-II and bacopasaponin-C by a previously described validated high performance liquid chromatography (HPLC) method [66]. The HPLC system consisted of double pumps (LC-20AT Shimadzu, Japan) with UV detector (SPD-20A Shimadzu, Japan) and column (Purospher C18, 250 mm × 4.6 mm × 4 μm particle size). Briefly, 5 mg of *Bacopa monnieri* extract was mixed with 5 mL of HPLC grade methanol, centrifuged for 10 min at 3000 rpm, filtered through a 0.45 μm filter and the filtered solution was then injected into the HPLC system. Mobile phase was prepared by mixing 0.2% phosphoric acid and acetonitrile (62:38 *v*/v), sonicated for 15 min and filtered under vacuum through a 0.45 μm filter paper. With the system flow rate set at 0.6 mL/min and the wavelength of the detector at 205 nm, all the peaks in *Bacopa monnieri* extract were obtained within a runtime of 33 min. The peaks in *Bacopa monnieri* extract were confirmed by spiking the standards with samples.

### Animals

Male Sprague-Dawley rats, weighing 300–450 g were used. They were maintained in a 12 h light/dark cycle at 22 ± 2 °C throughout the study duration with ad libitum access to food and water. All experimental procedures on animals were approved by the Ethical Committee (13/EC-15/Pharm) of the Department of Pharmacy, University of Peshawar, which were in accordance with the UK Animals (Scientific Procedures) Act 1986 and conformed to the ARRIVE guidelines for the reporting of in vivo experiments.

### Induction of neuropathic nociception in rats

The chronic constriction injury (CCI) model, which is based on a partial denervation of the sciatic nerve as described by Bennett and Xie [69] was used for induction of neuropathic nociception. The animals were anesthetized with an intraperitoneal injection of a mixture of xylazine (10 mg/kg) and ketamine (100 mg/kg). With proper surgical care, four loose ligatures were tied with a double knot, 1 mm apart and proximal to the trifurcation of the sciatic nerve. The constriction of nerve was minimal and was immediately stopped until a brief twitch was observed. An identical operation was performed, except that the sciatic nerve was not ligated in the sham-operated animals [70].

### Treatment groups

All drugs were dissolved in normal saline. *Bacopa monnieri* extract was orally administered daily in doses of 40 and 80 mg/kg [71, 72]. Gabapentin, used as a positive control, was intraperitoneally administered once daily at a dose of 75 mg/kg [73]. All animals were randomly divided into the following groups (*n* = 6 animals per group) and the study was continued for 21 days.

- Group 1: Sham-operated control
- Group 2: Chronic constriction injury (CCI) control
- Group 3: CCI + gabapentin (75 mg/kg/day, i.p)
- Group 4: CCI + *Bacopa monnieri* extract (40 mg/kg/day, p.o)
- Group 5: CCI + *Bacopa monnieri* extract (80 mg/kg/day, p.o)
- Group 6: Sham-operated + gabapentin (75 mg/kg/day, i.p)
- Group 7: Sham-operated + *Bacopa monnieri* extract (40 mg/kg/day, p.o)
- Group 8: Sham-operated + *Bacopa monnieri* extract (80 mg/kg/day, p.o)

### Neuropathic nociception testing paradigms

The behavioral testing paradigms of allodynia and hyperalgesia were used to assess neuropathic nociception. The animals were transferred to a specially designed elevated wire mesh bottom table. They were acclimatized for ~20 min and subsequently tested for static and dynamic allodynia, heat hyperalgesia, mechanical hyperalgesia and cold allodynia on −3 (pre-surgery) and post-surgically on days 3, 7, 14 and 21.

#### Static allodynia

The up and down method as reported by Chaplan et al. [74] was used for the assessment of static allodynia. The mid-plantar surface of the operated left hindpaw was subjected to a series of 8 von Frey filaments (0.4, 0.70, 1.20, 2.00, 3.63, 5.50, 8.50, and 15.10 g) (Stoelting USA). Each hair was applied perpendicularly until it buckled. A period of 6 s was selected as a cut-off time or until a positive response occurred (withdrawal of paw). The pattern of each response was converted to the 50% withdrawal threshold (PWT, g).

#### Dynamic allodynia

The mid-plantar surface of the operated left hindpaw was lightly stroked with a cotton bud. The time taken to show a withdrawal reaction (lifting or licking the paw) was considered as the paw withdrawal latency (PWL). A cut-off time of 15 s was selected [75].

#### Heat hyperalgesia

A heated plate maintained at a constant temperature (56 °C) was touched slightly with the mid-plantar surface of the operated left hindpaw. The heat source was adjusted at the beginning of the experiment to yield a paw flick in ∼10 s. The paw withdrawal latency (PWL) was recorded, with a minimal value of 0.5 s and a maximum of l0 s [76].

#### Punctate hyperlagesia

The tip of an ordinary safety pin was pressed against the skin of the mid-plantar surface of the operated left hindpaw such that the skin was dimpled but not penetrated. The duration of paw withdrawal (PWD) was recorded, with an arbitrary minimal time of 0.5 s (for the brief normal response) and a maximal cut-off of l5 s [77].

#### Cold allodynia

The mid-plantar surface of the operated left hindpaw was delicately sprayed with 50 μL of acetone using a needle connected to a syringe. The duration of the withdrawal response (PWD) was recorded with an arbitrary minimal value of 0.5 s and a maximum of 15 s [77].

### Statistical analysis

Data were expressed as mean ± S.E.M. The final data were analyzed using two-way repeated measures analysis of variance (ANOVA) followed by post hoc Bonferroni test. Student’s t test was used for statistical significance between pre-surgical and post-surgical response data. All statistical analyses were conducted using GraphPad Prism 5 (GraphPad Software Inc. San Diego CA, USA). A *P* value of ≤0.05 was accepted as significant.

## Results

### Standardization of *Bacopa monnieri* methanolic extract

**Fig. 1:**
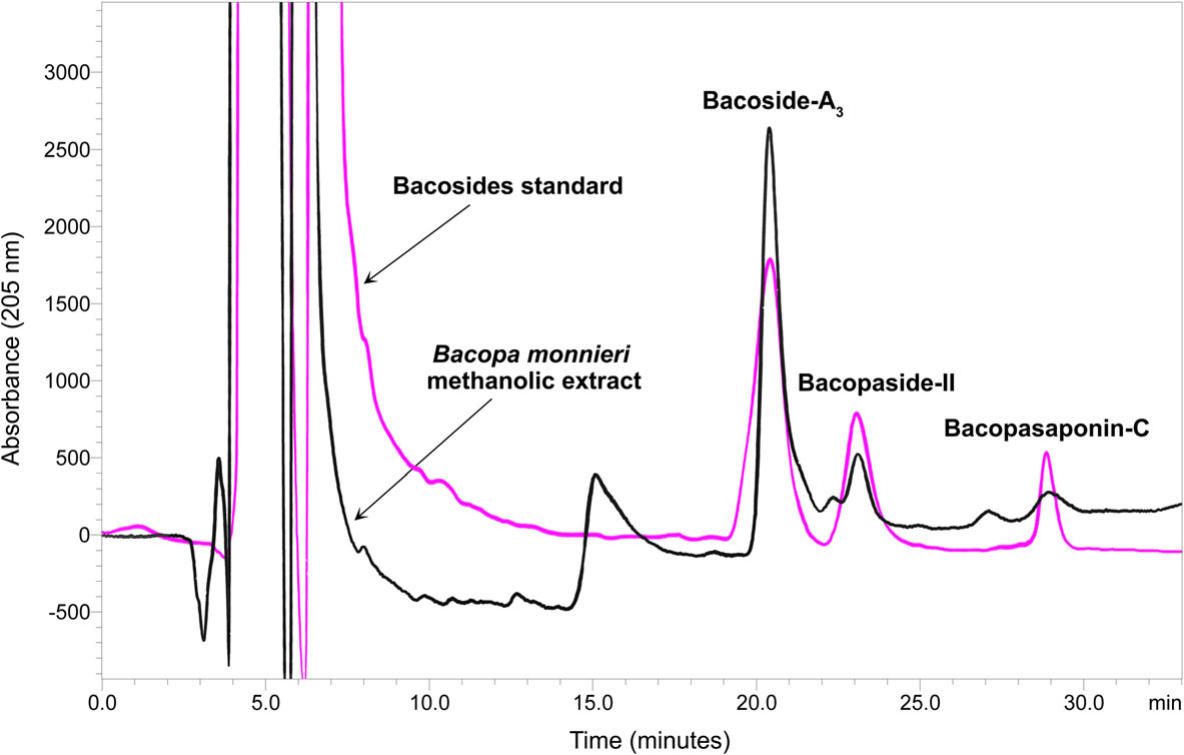
HPLC-UV fingerprint of bacoside “A” major components (bacoside-A_3_, bacopaside-II and bacopasaponin-C) quantified in *Bacopa monnieri* methanolic extract overlaid with that of standard bacosides

The HPLC-UV analysis of the *Bacopa monnieri* methanolic extract confirmed the presence of bacoside-A_3_, bacopaside-II and bacopasaponin-C in quantities of 31.62 μg/mg, 5.41 μg/mg and 1.01 μg/mg respectively (Fig. 1). The chromatographic analysis showed that bacoside-A_3_ was the major component of bacoside-A in the *Bacopa monnieri* extract. The total quantity of bacoside “A” three-major components was revealed as 38.04 μg/mg of extract.

### Effect of *Bacopa monnieri* on mechanical allodynia

#### Effect of *Bacopa monnieri* on static mechano-allodynia

A significant decrease (*P* < 0.001) in the nociceptive threshold to the innocuous mechanical von Frey hairs (static allodynia) was produced in the operated left hindpaw after unilateral sciatic nerve ligation, when observed on days 3–21 as compared to the sham-operated animals. Additionally, the unilateral ligation caused a significant reduction in PWT, prominent on day 3 (5.037 ± 0.7251 g, *P* < 0.01), day 7 (2.262 ± 0.3148 g, *P* < 0.001), day 14 (1.872 ± 0.3543 g, *P* < 0.001) and day 21 (1.538 ± 0.5192 g, *P* < 0.001) compared to their pre-surgery baseline values (14.42 ± 0.9910 g).

The treatments have significant main dose effect on the CCI-induced static allodynia [time = (*F* (4175) = 22.79, *P* < 0.0001), treatment = (*F* (7175) = 48.34, *P* < 0.0001), interaction = (*F* (28,175) = 3.24, *P* < 0.0001)]. Daily administration of *Bacopa monnieri* in doses of 40 and 80 mg/kg alleviated the evoked nociception to normally non-painful gentle static pressure stimuli on the hindpaw mid-plantar surface by significantly restoring (*P* < 0.01, *P* < 0.001) the CCI-induced decrease in PWT, noticed on days 3 (only for 80 mg/kg), 7, 14 and 21 post-ligation. The PWT values for the 40 mg/kg dose on day 3 (8.215 ± 1.445 g, *P* < 0.01), day 7 (7.237 ± 0.4558 g, *P* < 0.001), day 14 (8.138 ± 1.011 g, *P* < 0.01) and day 21 (8.557 ± 1.583 g, *P* < 0.01) approached the pre-surgery baseline values (15.00 g). Similarly, the 80 mg/kg dose also afforded a highly efficient protective effect as the post-surgery PWT values on days 3 (9.682 ± 1.393 g, *P* < 0.05), 7 (11.85 ± 1.501 g), 14 (10.55 ± 1.413 g, *P* < 0.05) and 21 (11.46 ± 1.357 g, *P* < 0.05) drew close to pre-ligation PWT of 15.00 g.

The daily 75 mg/kg dose of the positive control, gabapentin produced a robust static anti-allodynic effect by significantly relieving (*P* < 0.01, *P* < 0.001) the neuropathic nociception evoked by application of von Frey hairs to the mid-plantar surface of the operated left hindpaw compared to the untreated CCI control group. The static anti-allodynic effect was also evidenced by a less significant difference in PWT from pre-surgery value of 15.47 ± 0.4683 g (day −3) to post-surgery values of 10.26 ± 1.714 g (day 3, *P* < 0.05), 9.737 ± 2.213 g (day 7), 10.72 ± 1.290 g (day 14, *P* < 0.01) and 10.11 ± 1.659 g (day 21, *P* < 0.05).

**Fig. 2:**
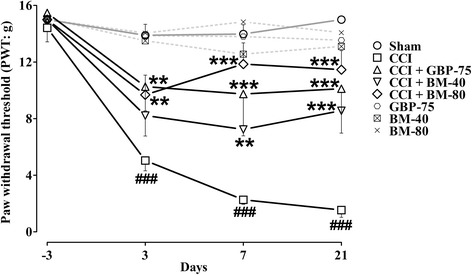
Effect of *Bacopa monnieri* methanolic extract (BM-40 and BM-80) and gabapentin (GBP-75) on the maintenance of chronic constriction injury (CCI) induced static allodynia (diminished von Frey filament threshold pressure; PWT in g). Values expressed as mean ± S.E.M. ^###^ *P* < 0.001 compared to sham-operated animals, ** *P* < 0.01, *** *P* < 0.001 compared to CCI-operated untreated animals, two-way repeated measures ANOVA followed by post hoc Bonferroni analysis. *n* = 6 rats per group

No static allodynia was observed in the sham-operated control or sham plus *Bacopa monnieri*/gabapentin treated animals at any time during the 30 days of observation (Fig. 2).

#### Effect of *Bacopa monnieri* on dynamic mechano-allodynia

In comparison to the sham-operated animals, the dynamic allodynia in the CCI-operated animals took a slow course to develop and was observable on day 14 when a brisk reduction (*P* < 0.001) in the latency to withdraw the operated left hindpaw was produced after lightly stroking the mid-plantar region with a cotton bud. The reduction in the PWL to normally non-painful light-pressure moving stimuli was noticeable for the subsequent week of study (day 21, *P* < 0.001). Moreover, the PWL values also significantly changed from the pre-surgery baseline PWL values of 11.85 ± 1.426 s to 3.110 ± 0.4216 s (*P* < 0.01) and 3.515 ± 0.6215 s (*P* < 0.01) on days 14 and 21, respectively.

Treatment with *Bacopa monnieri* and gabapentin significantly modified the CCI-induced dynamic allodynia [time = (*F* (4175) = 3.00, *P* < 0.0378), treatment = (*F* (7175) = 9.81, *P* < 0.0001), interaction = (*F* (28,175) = 1.76, *P* < 0.0152)]. *Bacopa monnieri* treated animals (40 and 80 mg/kg), significantly elevated (*P* < 0.01, *P* < 0.001) the CCI-induced reduction in PWL on days 14 and 21. The resistance against hyper-responsiveness to the innocuous stroking of cotton bud was also evident by a non-significant difference in the latency to lift the operated hindpaw on day 14 (8.605 ± 1.584 s and 12.91 ± 1.327 s) and day 21 (11.93 ± 1.451 s and 11.97 ± 1.242 s) compared to day −3 (12.60 ± 1.636 s and 14.51 ± 0.3392 s), respectively at doses of 40 and 80 mg/kg.

The daily systemic dose of the positive control, gabapentin (75 mg/kg) showed a dominant protective effect against CCI-induced evoked dynamic allodynia on days 14 and 21 (*P* < 0.001), which was further confirmed by elevation of post-surgery PWL on days 14 (11.07 ± 1.099 s) and 21 (12.02 ± 1.161 s) compared to that of pre-surgery value on day −3 (11.61 ± 1.165 s).

**Fig. 3:**
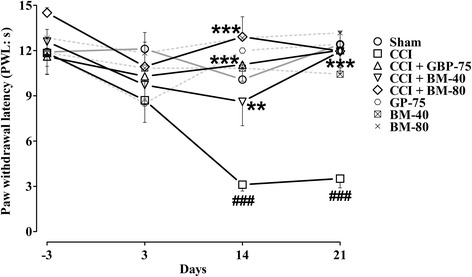
Effect of *Bacopa monnieri* methanolic extract (BM-40 and BM-80) and gabapentin (GBP-75) on the maintenance of chronic constriction injury (CCI) induced dynamic allodynia (diminished paw withdrawal latency to light brushing; PWL in s). Values expressed as mean ± S.E.M. ^###^ *P* < 0.001 compared to sham-operated animals, ** *P* < 0.01, *** *P* < 0.001 compared to CCI-operated untreated animals, two-way repeated measures ANOVA followed by post hoc Bonferroni analysis. *n* = 6 rats per group

The sham-operated control as well as the per se treated *Bacopa monnieri*/gabapentin animals did not show any dynamic allodynia during the entire study duration (Fig. 3).

### Effect of *Bacopa monnieri* on heat hyperalgesia

A significant exaggerated (*P* < 0.001) response to normally non-painful heat stimuli evoked nociception was observed post-surgically in the untreated CCI control animals as compared to the sham-operated animals during the entire study duration. The nociceptive thermal sensation in the mid-plantar area measured as reduced latency to paw withdrawal was significantly (*P* < 0.001) changed from pre-surgery baseline of 5.797 ± 0.2119 s to 2.032 ± 0.1028 s, 1.938 ± 0.07910 s, 1.932 ± 0.1795 s and 1.948 ± 0.1477 s on days 3, 7, 14 and 21, respectively.

Significant effect on the CCI-induced heat hyperalgesia [time = (*F* (4175) = 15.84, *P* < 0.0001), treatment = (*F* (7175) = 43.21, *P* < 0.0001), interaction = (*F* (28,175) = 2.92, *P* < 0.0001)] was produced by *Bacopa monnieri* and gabapentin. Treatment with *Bacopa monnieri* at 40 and 80 mg/kg altered the CCI-induced exaggerated response by significantly elevating (*P* < 0.05, *P* < 0.01) the PWL upon heat stimuli starting from day 7 till the end of study (day 21). Both the 40 and 80 mg/kg doses uplifted the post-surgical PWL on day 7 (3.787 ± 0.5617 s and 4.305 ± 0.8559 s), day 14 (3.705 ± 0.3627 s, *P* < 0.05 and 3.833 ± 0.5276 s, *P* < 0.05) and day 21 (3.750 ± 0.2884 s, *P* < 0.05 and 4.235 ± 0.3723 s, *P* < 0.01), respectively when compared to their pre-surgical PWL values (5.838 ± 0.5921 s and 6.767 ± 0.5032 s).

A highly efficient resistance (*P* < 0.05, *P* < 0.01) to heat hyperalgesia was produced by a daily intraperitoneal dose of gabapentin (75 mg/kg) on days 3–21 as compared to the CCI control group. The post-ligation threshold to perceive heat stimuli evoked nociception was elated by gabapentin to 3.587 ± 0.2387 s (*P* < 0.05) (day 3), 3.910 ± 0.3705 s (*P* < 0.01) (day 7), 3.933 ± 0.2500 s (*P* < 0.01) (day 14) and 4.395 ± 0.2487 s (day 21), compared to pre-ligation baseline of 5.770 ± 0.3662 s (day −3).

**Fig. 4:**
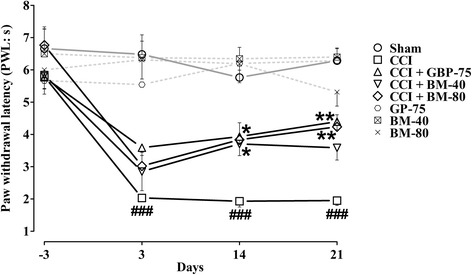
Effect of *Bacopa monnieri* methanolic extract (BM-40 and BM-80) and gabapentin (GBP-75) on the maintenance of chronic constriction injury (CCI) induced heat hyperalgesia (shortened paw withdrawal latency to heat stimulus; PWL in s). Values expressed as mean ± S.E.M. ^###^ *P* < 0.001 compared to sham-operated animals, * *P* < 0.05, ** *P* < 0.01 compared to CCI-operated untreated animals, two-way repeated measures ANOVA followed by post hoc Bonferroni analysis. *n* = 6 rats per group

The per se treatment with *Bacopa monnnieri* and gabapentin treated animals showed a robust anti-hyperalgesic effect (*P* < 0.001) throughout the experimental duration as compared to the CCI controls (Fig. 4).

### Effect of *Bacopa monnieri* on punctate hyperalgesia

The unilateral sciatic nerve ligation produced an exaggerated response to a noxious stimuli evoked nociception manifested as a significant increase (*P* < 0.001) in PWD, which was prominent from day 3 up to day 21. The hyperalgesia induced by manual pricking of the mid-plantar surface of the operated left hindpaw significantly raised (*P* < 0.01, *P* < 0.001) the PWD from pre-surgery baseline of 0.5 s to 8.707 ± 0.9516 s on day 3, 10.37 ± 1.760 s on day 7, 8.278 ± 1.598 s on day 14 and 8.995 ± 0.6923 s on day 21 .

Analysis revealed significant main dose effect was afforded by *Bacopa monnieri* and gabapentin on the CCI-induced mechanical hyperalgesia [time = (*F* (4175) = 23.93, *P* < 0.0001), treatment = (*F* (7175) = 103.35, *P* < 0.0001), interaction = (*F* (28,175) = 7.06, *P* < 0.0001)]. Treatment with *Bacopa monnieri* extract significantly abolished (*P* < 0.001) the CCI-induced hyperalgesic response to the noxious pin prick at doses of 40 and 80 mg/kg during the entire treatment duration. The extract significantly counterbalanced (*P* < 0.05, *P* < 0.01) the CCI-induced increased PWD upon application of a noxious stimulus at 40 and 80 mg/kg on day 3 (4.702 ± 1.458 s and 3.060 ± 0.4325 s), day 7 (2.985 ± 0.3177 s and 3.038 ± 0.6042 s), day 14 (2.925 ± 0.4848 s and 2.790 ± 0.3451 s) and day 21 (2.833 ± 0.3955 s and 3.167 ± 0.4609 s) compared to their respective pre-surgery baseline PWD (0.5 s).

The hypersensitivity during the experimental period was also significantly lessened (*P* < 0.001) by the 75 mg/kg daily dose of gabapentin compared to the untreated CCI control group. Moreover, in comparison to the baseline (0.5 s, day −3), the positive control also opposed the sharp pricking hyper-responsiveness when tested post-surgically for PWD on day 3 (4.088 ± 0.5006 s, *P* < 0.001), day 7 (3.955 ± 0.2851 s, *P* < 0.001), day 14 (3.218 ± 0.3680 s, *P* < 0.001) and day 21 (3.180 ± 0.2998 s, *P* < 0.001).

**Fig. 5:**
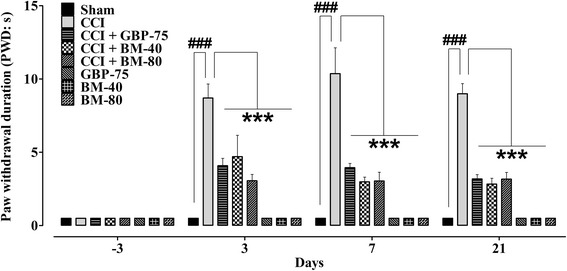
Effect of *Bacopa monnieri* methanolic extract (BM-40 and BM-80) and gabapentin (GBP-75) on the maintenance of chronic constriction injury (CCI) induced punctate hyperalgesia (increased paw withdrawal duration to pin prick; PWD in s). Values expressed as mean ± S.E.M. ^###^ *P* < 0.001 compared to sham-operated animals, *** *P* < 0.001 compared to CCI-operated untreated animals, two-way repeated measures ANOVA followed by post hoc Bonferroni analysis. *n* = 6 rats per group

Significant (*P* < 0.001) alleviation of punctate hyperalgesia was demonstrated by the sham-operated plus *Bacopa monnieri* and gabapentin treated animals throughout the treatment period (Fig. 5).

### Effect of *Bacopa monnieri* on cold allodynia

A significant exaggerated withdrawal reflex (*P* < 0.001) hastened upon application of a drop of acetone on the mid-plantar surface of the operated left hindpaw prompted by unilateral sciatic nerve ligation compared to that produced by sham-operation. The duration of paw lifting incited by the cooling effect of acetone was markedly increased (*P* < 0.001) to 9.948 ± 1.200 s, 12.52 ± 0.818 s, 12.23 ± 0.784 s and 14.22 ± 0.438 s on days 3, 7, 14 and 21, respectively from the pre-surgery baseline of 0.5 s (day −3).

*Bacopa monnieri* and gabapentin have significant effect on the maintenance of CCI-induced cold allodynia [time = (*F* (4175) = 96.57, *P* < 0.0001), treatment = (*F* (7175) = 255.61, *P* < 0.0001), interaction = (*F* (28,175) = 18.62, *P* < 0.0001)]. The normally non-painful cold stimuli evoked nociception initiated by the ligation procedure was significantly subsided (*P* < 0.001) by *Bacopa monnieri* in doses of 40 and 80 mg/kg for the entire study period. The increment in paw withdrawal duration after surgery was suppressed by 40 mg/kg to 6.567 ± 1.149 s (day 3, *P* < 0.01), 5.040 ± 0.5520 s (day 7, *P* < 0.001), 5.568 ± 0.3626 s (day 14, *P* < 0.001) and 5.595 ± 0.5726 s (day 21, *P* < 0.001) when compared to pre-surgery value of 0.5 s. Likewise, the decrement in PWD was also prominent for 80 mg/kg on day 3 (3.008 ± 0.1528 s, *P* < 0.001), day 7 (4.148 ± 0.1946 s, *P* < 0.001), day 14 (5.380 ± 0.5755 s, *P* < 0.001) and day 21 (5.918 ± 1.013 s, *P* < 0.01).

A robust increase (*P* < 0.001) in the threshold to perceive the acetone-induced nociceptive cold stimuli was demonstrated by the positive control, gabapentin when administered daily in a dose of 75 mg/kg. The increase in nociceptive threshold was prominent when the post-surgical duration of paw withdrawal was compared to pre-surgery baseline of 0.5 s (day −3) and measured as 4.657 ± 0.5292 s on day 3 (*P* < 0.001), 5.252 ± 0.7290 s on day 7 (*P* < 0.01), 6.152 ± 0.8892 s on day 14 (*P* < 0.01) and 3.870 ± 0.6106 s on day 21 (*P* < 0.01).

**Fig. 6:**
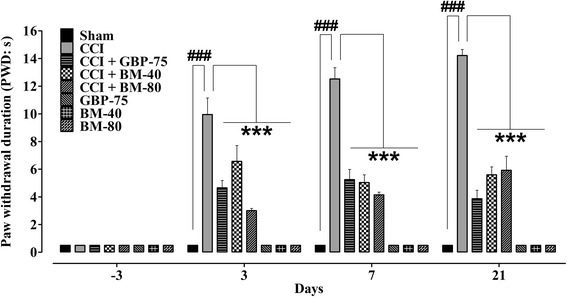
Effect of *Bacopa monnieri* methanolic extract (BM-40 and BM-80) and gabapentin (GBP-75) on the maintenance of chronic constriction injury (CCI) induced cold allodynia (increased paw withdrawal duration to a drop of acetone; PWD in s). Values expressed as mean ± S.E.M. ^###^ *P* < 0.001 compared to sham-operated animals, *** *P* < 0.001 compared to CCI-operated untreated animals, two-way repeated measures ANOVA followed by post hoc Bonferroni analysis. *n* = 6 rats per group

The sham-operated plus *Bacopa monnier*a/gabapentin treated animals significantly attenuated (*P* < 0.001) the acetone-evoked cold allodynia throughout the study period, compared to the CCI controls (Fig. 6).

## Discussion

This study assessed the beneficial effect of *Bacopa monnieri* in the chronic constriction injury model of neuropathic pain under standardized experimental conditions. Animal models of peripheral nerve injury combined with testing of pain hypersensitivity are useful in representing human neuropathic pain syndromes [78]. Therefore, new drugs need be evaluated in these animal models before going into clinical trials for their utility in neuropathic pain. The chronic sciatic nerve constriction injury model mimics many of the pathophysiological properties of chronic neuropathic pain in human and has been demonstrated to be sensitive to a number of compounds which are used clinically for the symptomatic treatment of chronic neuropathic pain. In this study, the time-course of development and the degree of mechanical allodynia and hyperalgesia in the employed CCI method was similar as previously reported elsewhere [79].

Chronic pain following peripheral or central nerve injury presents more difficult task as the drugs successfully used in an acute pain are usually inefficient for neuropathic pain. This study has demonstrated that treatment with *Bacopa monnieri* significantly suppressed the evoked nociceptive responses when orally administered daily in doses of 40 and 80 mg/kg, which were tolerable and benign based on the toxicity profile [31, 80]. Similar doses of *Bacopa monnieri* have been shown to possess significant neuroprotective effects including its ability to protect the cerebral cortex [81], enhance dendritic length and arborization of amygdala [71] and hippocampal CA_3_ neurons [72]. Moreover, these doses also have significant anti-stress and anti-depressant activities [38, 82] and neuropathic pain is often associated with comorbidities such as anxiety and depression, resulting in a low health-related quality of life [83].

In this study, we tested *Bacopa monnieri* against allodynia and hyperalgesia induced after a peripheral nerve injury. Allodynia and hyperalgesia are frequent symptoms of disease and may be useful adaptations to protect vulnerable tissues. Both may, however, also emerge as diseases in their own right [84]. A number of compounds have been used to modulate neuropathic allodynia, hyperalgesia and other manifestations of neuropathic pain [73, 85–87]. In this study, we clearly demonstrated that *Bacopa monnieri* succinctly inhibited the maintenance of static and dynamic allodynia, heat, punctate and cold allodynia in the rat model of CCI-induced neuropathic nociception. A previous study from the same laboratory showed that a hydroethanolic extract of *Bacopa monnieri* (80 mg/kg, i.p) increased the hot-plate nociceptive response latency, in mice [88]. However, in this study, the tested doses of methanolic *Bacopa monnieri* extract have demonstrated no effect on the latency time in the sham-operated control rats. This discrepancy in the results might be due to differences in the paradigms concerning the sensitivity of response latency in the heat-nocifensive test adopted in this study (see Additional file 1) and that of the hot-plate test [89–91] as used previously in the same laboratory [88]. Additionally, the effect of variations in bacosides contents, present in the hydroethanolic and methanolic extracts cannot be ignored, as bacoside-A concentration varies between different solvents [46]. Moreover, a significant variation exists in the bacoside-A content of the various *Bacopa monnieri* accessions collected from different locations [64, 92].

The neuropathic nociception alleviating effect afforded by *Bacopa monnieri* was equipotent to the positive control, gabapentin which was daily administered in a dose of 75 mg/kg for 21 days. Gabapentin is currently recommended as first-line treatment for neuropathic pain and is effective at relieving allodynia and hyperalgesia not only in animal models, but also in numerous clinical trials in a wide variety of pain syndromes including diabetic neuropathy and postherpetic neuralgia [93–95]. However, its clinical effectiveness is limited to the magnitude of pain relieved and to the occurrence of dose-dependent side effects, of which dizziness, somnolence, ataxia and lethargy, being the most significant [93].

Neuropathic pain is the consequence of a complex interplay of mechanisms in the peripheral and central nervous system which include: 1) Sensitization of the nociceptor (increase bradykinin, prostaglandins, serotonin, substance P, NGF). 2) Abnormal ectopic excitability of afferent neurons and sympathetically maintained pain (voltage-gated sodium and calcium channels). 3) Disinhibition of nociception at the spinal inhibitory network (GABAergic, opioidergic and glycinergic receptors). 4) Pronociceptive facilitation at the spinal dorsal horn (glutaminergic receptors, TNFα, NGF, microglia). 5) Supraspinal mechanisms (decrease in norepinephrine, serotonin, dopamine, endogenous opioids). Treatment based on the mechanism(s) of pain is widely accepted to be theoretically better than treatment based on the cause of pain [96]. Although, the mechanism through which *Bacopa monnieri* ameliorates allodynia and hyperalgesia is still unknown; nonetheless, in view of its myriad pharmacological profile it can be speculated that multiple mechanisms might be involved as *Bacopa monnieri* possesses anti-nociceptive activity [30] mediated through opioidergic mechanisms [88], enhances the morphine anti-nociceptive effects [66], increases synaptic plasticity [97], up-regulates vesicular glutamate transporter type 2 (VGLUT2) [98] and decreases the immunodensity of glutamate/*N*-methyl-D- aspartate receptor subtype 1 (NMDAR1) [99]. What is more, *Bacopa monnieri* increases GABA, GABA_A_ receptor subunit, GABA_A_ receptor binding and up-regulation of GAD gene [11]. Additionally, *Bacopa monnieri* decreases the release of TNF-α and IL-6 [22] and inhibits the activities of COX-2, LOX-5 and LOX-15 [21]. Herbal medicines are reported to be beneficial in the management of painful neuropathy [100–102] and recently there has been a dramatic increase in the use of complementary and alternative medicine especially herbal therapies, to reduce pain [103–105].

## Conclusions

In summary, this study demonstrates for the first time that a bacosides rich fraction of *Bacopa monnieri* presents marked antinociceptive properties by alleviating allodynia and hyperalgesia in the chronic constriction injury model of neuropathic pain in rats. *Bacopa monnieri* may constitute a beneficial herbal remedy for the efficient management of neuropathic pain syndromes. The antinociceptive effect of *Bacopa monnieri* against neuropathic pain requires further studies not only for elucidating the exact mechanism but also needs to be tested in other neuropathic pain models as there are differences between animal models in terms of the magnitude of each pain component. Additionally, the major bioactive constituent, bacoside-A should be tested in the CCI model, to validate its role in the neuropathic pain alleviating effect of *Bacopa monnieri*.

## Additional file

Additional file 1: Effect of acute administration of *Bacopa monnieri* extract on heat-nocifensive response latency evoked by a heated probe maintained either at a constant temperature of 56 °C with a cut-off latency time of 10 s (Experiment S1) or at 50 °C with a cut-off latency time of 60 s (Experiment S2) in normal rats. (DOCX 516 kb)

## Abbreviations

BM: *Bacopa monnieri* methanolic extract
CCI: Chronic constriction injury
COX: Cyclooxygenase
GABA: Gamma amino butyric acid
GAD: Glutamic acid decarboxylase
GBP: Gabapentin
HPLC: High performance liquid chromatography
IL-6: Interleukin 6
LOX: Lipoxygenase
NGF: Nerve growth factor
NMDAR1: Glutamate/*N*-methyl-D- aspartate receptor subtype 1
PWD: Paw withdrawal duration
PWL: Paw withdrawal latency
PWT: Paw withdrawal threshold
TNFα: Tumor necrosis factor alpha
VGLUT2: Vesicular glutamate transporter
