# Supplementary material for: A bacosides containing Bacopa monnieri extract alleviates allodynia and hyperalgesia in the chronic constriction injury model of neuropathic pain in rats
Source: BMC Complement Altern Med. 2017 Jun 5;17:293. doi: 10.1186/s12906-017-1807-z (PMC5460461; doi:10.1186/s12906-017-1807-z)
Supplement: Additional file 1: — Effect of acute administration of Bacopa monnieri extract on heat-nocifensive response latency evoked by a heated probe maintained either at a constant temperature of 56 °C with a cut-off latency time of 10 s (Experiment S1) or at 50 °C with a cut-off latency time of 60 s (Experiment S2) in normal rats. (DOCX 516 kb) [file 12906_2017_1807_MOESM1_ESM.docx]

**Additional file 1**

**A bacosides containing *Bacopa monnieri* extract alleviates allodynia and hyperalgesia in the chronic constriction injury model of neuropathic pain in rats**

Muhammad Shahid^1,2^, Fazal Subhan^1^, Nisar Ahmad^1^, Ihsan Ullah^3^

**Affiliations**

^1^Department of Pharmacy, University of Peshawar, Peshawar, Pakistan

^2^Department of Pharmacy, Sarhad University of Science and Information Technology, Peshawar, Pakistan

^3^Department of Pharmacy, University of Swabi, Swabi, Pakistan

**Email addresses**

Muhammad Shahid: shahidsalim_2002@hotmail.com

Fazal Subhan: fazal_subhan@upesh.edu.pk

Nisar Ahmad: nisarahmadsatal@yahoo.com

Ihsan Ullah: ihsanmkd@gmail.com

**Correspondence**

Professor Fazal Subhan

Department of Pharmacy

University of Peshawar

Peshawar-25120

Khyber Pakhtunkhwa, Pakistan

Email: fazal_subhan@upesh.edu.pk

Muhammad Shahid

shahidsalim_2002@hotmail.com

**Effect of acute administration of *Bacopa monnieri* extract on heat-nocifensive response latency in normal rats**

The following additional experiments were performed to address the discrepancy in the results of heat-nocifensive response latency between the present study (as observed for the sham-operated plus *Bacopa monnieri* treated rats) and a previous study on *Bacopa monnieri* in the mouse hot-plate test paradigm conducted in the same laboratory [1].

**Materials**

*Bacopa monnieri* methanolic extract was used which was prepared and standardized as mentioned in the manuscript. Morphine was obtained through proper channel from Punjab Drug House, Lahore, Pakistan. *Bacopa monnieri* extract and morphine were dissolved in normal saline, which was locally purchased (Zeesol NS, Shahzaib Pharmaceuticals Pvt. Ltd. Haripur Pakistan).

**Animals**

Male Sprague-Dawley rats, weighing 200-250 g were used. They were maintained in a 12 h light/dark cycle at 22 ± 2 ^o^C with *ad libitum* access to food and water. The experimental procedures on animals were approved by the Ethical Committee (13/EC-15/Pharm) of the Department of Pharmacy, University of Peshawar, which were in accordance with the UK Animals (Scientific Procedures) Act 1986 and conformed to the ARRIVE guidelines for the reporting of *in vivo* experiments.

**Methods**

A total of 36 animals were randomly assigned to 6 groups (*n* = 6 rats per group). Half of those (i.e. 3 groups) were used for experiment 1 and the other half were used for experiment 2. The experimental protocol for both the experiments is as follows:

***Experiment S1***

Group 1: Saline-treated control (5 mL/kg, p.o), *n* = 6

Group 2: *Bacopa monnieri* extract (80 mg/kg, p.o) *n* = 6

Group 3: Morphine (5 mg/kg, i.p) *n* = 6

The animals were treated with saline (5 mL/kg, p.o), *Bacopa monnieri* extract (80 mg/kg, p.o) and morphine (5 mg/kg, i.p), which was used as positive control. After 1 h (for *Bacopa monnieri* extract) or 20 min (for morphine), the animals were tested for response latencies using a heated probe maintained at a constant temperature of 56^o^C with a cut-off latency time of 10 s.

***Experiment S2***

Group 1: Saline-treated control (5 mL/kg, p.o), *n* = 6

Group 2: *Bacopa monnieri* (80 mg/kg, p.o) *n* = 6

Group 3: Morphine (5 mg/kg, i.p) *n* = 6

The animals were similarly treated and tested as that of experiment 1 except that the response latencies were checked at a constant temperature of 50^o^C with a cut-off latency time of 60 s.

**Results and discussion**

In the experiment 1, acute administration of *Bacopa monnieri* extract did not produce any discernable antinociceptive effect as the mean response latencies (7.0 ± 0.57) were not significantly different from saline-treated controls (6.02 ± 0.32) (Figure 1S). However, in the experiment 2, significant attenuation (*P* < 0.01) of the acute phasic thermal nociception was demonstrated by *Bacopa monnieri* extract (80 mg/kg) (Figure S2). The positive control, morphine (5 mg/kg) produced significant changes in the nociceptive threshold in both experiment 1 and 2, as the heat-evoked response latencies were significantly different (*P* < 0.05 and *P* < 0.001) from that of saline-treated controls.


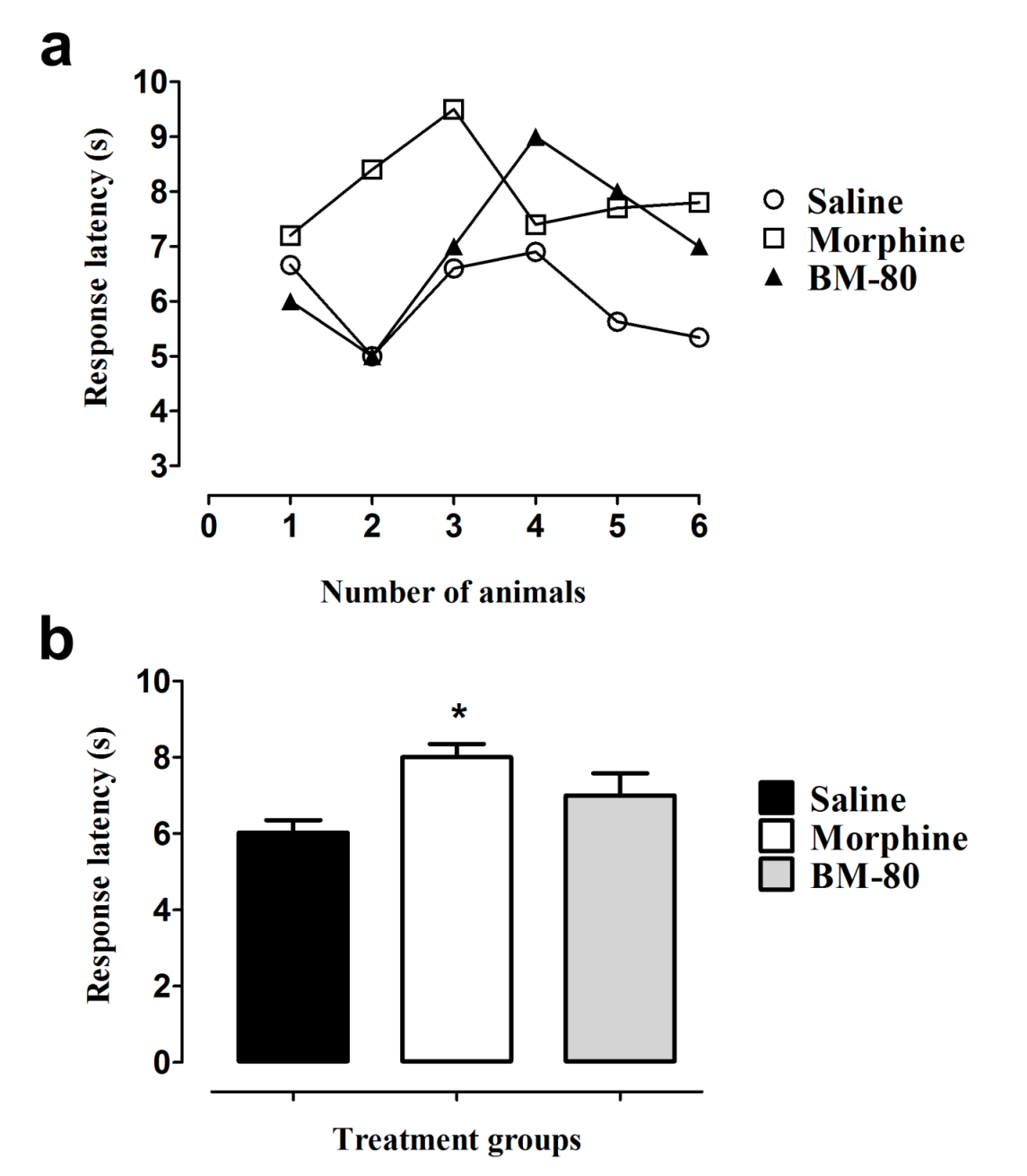


**Figure S1:** Effect of *Bacopa monnieri* at 80 mg/kg (BM-80), morphine at 5 mg/kg on the response-latency in the rat paw-withdrawal test, thermally evoked by a heated probe maintained at a constant temperature of 56°C with a validated cut-off latency time of 10 s. Paw response latencies for (**a**) individual rats; and (**b**) treatment groups with each bar represents mean endurance latency ± S.E.M. ^*^*P* < 0.05 as compared to saline-treated group, one-way ANOVA followed by *post hoc* Dunnett’s test, *n* = 6 rats per group.


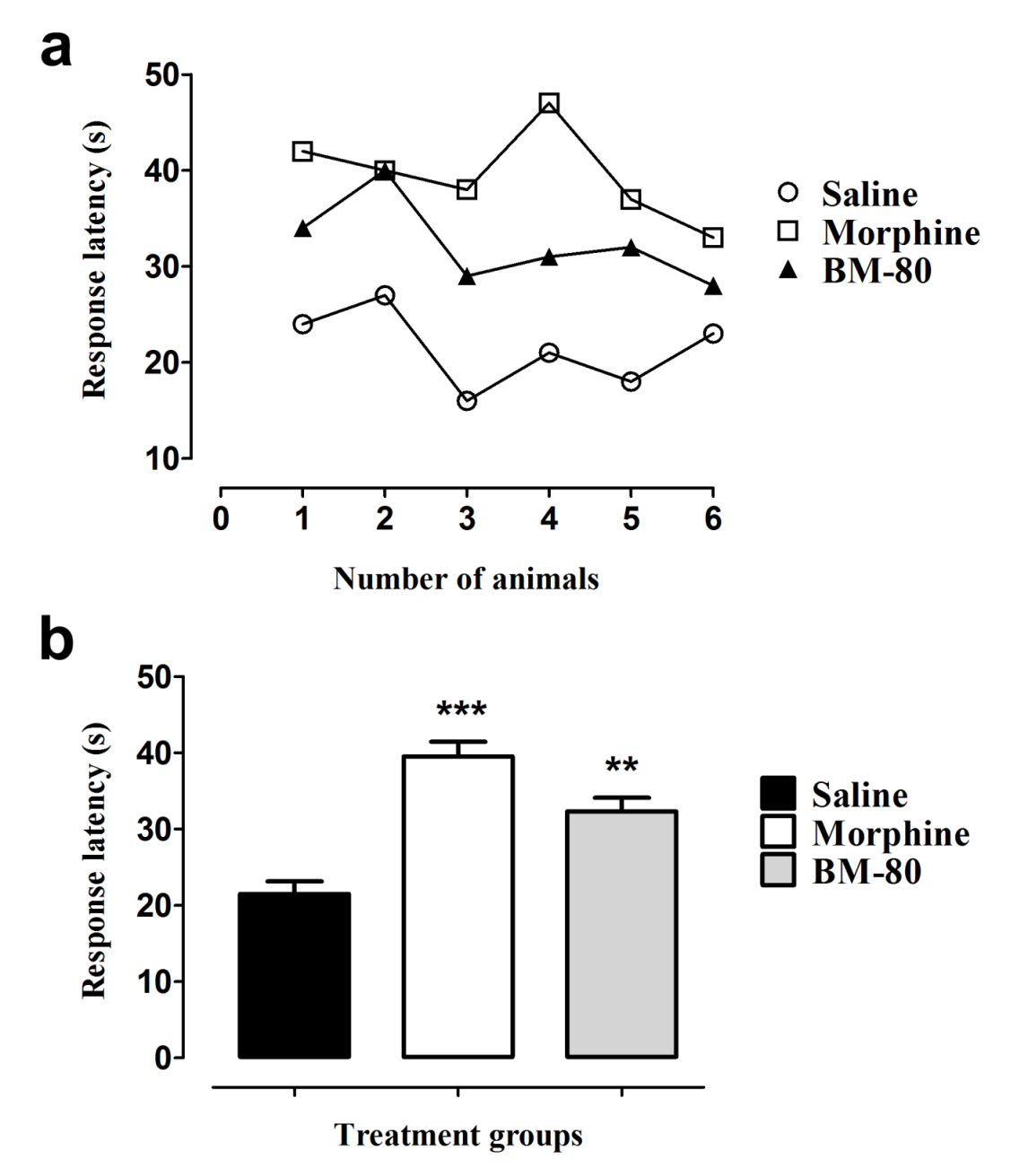


**Figure S2:** Effect of *Bacopa monnieri* at 80 mg/kg (BM-80), morphine at 5 mg/kg on the response-latency in the rat paw-withdrawal test, thermally evoked by a heated probe maintained at a constant temperature of 50°C with a validated cut-off latency time of 60 s. Paw response latencies for (**a**) individual rats; and (**b**) treatment groups with each bar represents mean endurance latency ± S.E.M. ^**^*P* < 0.01, ^***^*P* < 0.001 as compared to saline-treated group, one-way ANOVA followed by *post hoc* Dunnett’s test, *n* = 6 rats per group.

These results demonstrate that variations in temperature and cut-off latency time have significant effect on the expression of analgesic effect of *Bacopa monnieri* extract and that of the opioid agonist, morphine in the current paradigm. A previous study in rats has shown that lower hot-plate temperatures are much more sensitive to the effects of the opioid, morphine. The effect of a low dose of morphine (3 mg/kg) could be reliably detected with only 8 rats at 50°C, while the same dose would not be detected reliably at 55°C unless more than 55 rats are tested [2]. Similarly, lower hot-plate temperatures have been shown to be more sensitive to the effects of mild analgesics [3-4]. Additionally, the animal’s latency to respond also varies with the cut-off latency time. Typically, in mice it take 40 to 60 s to exhibit the response measure of interest in the hot-plate test, with a lower cut-off latency makes detection of drug-effects (e.g., dose response) difficult [5]. The results observed in the additional experiments corroborated these previous studies, in which lowering of temperature and prolongation of cut-off latency time caused the antinociceptive effect of *Bacopa monnieri* discernable, which was in relation to the effect of opioid agonist, morphine, when tested in the similar paradigm.

The results of these additional experiments clearly explain the inability of the tested doses of *Bacopa monnieri* (40 and 80 mg/kg) to produce any detectable antinociceptive effect in the sham-operated control rats, when examined for heat-hyperalgesia in a paw-withdrawal test, thermally evoked by a heated probe maintained at a constant temperature of 56°C with a validated cut-off latency time of 10 s. The results of these additional experiments also explain the potential analgesic property of *Bacopa monnieri* reported previously in the same laboratory in which the hydroethanolic extract of *Bacopa monnieri* (80 mg/kg, i.p) increased the response latency in a mouse hot-plate model using a metallic-plate maintained at a temperature of 54 ^o^C with a cut-off time of 30 s [1].

**References**

1. Subhan F, Abbas M, Rauf K, Arfan M, Sewell RD, Ali G. The role of opioidergic mechanism in the activity of *Bacopa monnieri* extract against tonic and acute phasic pain modalities. Pharmacologyonline. 2010;3:903-14.

2. Plone MA, Emerich DF, Lindner MD. Individual differences in the hotplate test and effects of habituation on sensitivity to morphine. Pain. 1996;66(2):265-70.

3. Ankier SI. New hot plate tests to quantify antinociceptive and narcotic antagonist activities. Eur J Pharmacol. 1974;27(1):1-4.

4. O'Callaghan JP, Holtzman SG. Quantification of the analgesic activity of narcotic antagonists by a modified hot-plate procedure. J Pharmacol Exp Ther. 1975;192(3):497-505.

5. Malmberg AB, Bannon AW. Models of nociception: hot-plate, tail-flick, and formalin tests in rodents. Current protocols in neuroscience. 1999:Chapter 8:Unit .9. doi: 10.1002/0471142301.ns0809s41.
